# Treatment of progressive supranuclear palsy with autologous adipose tissue-derived mesenchymal stem cells: a case report

**DOI:** 10.1186/1752-1947-8-87

**Published:** 2014-03-04

**Authors:** Soo Won Choi, Kwon Byong Park, Sang Kyu Woo, Sung Keun Kang, Jeong Chan Ra

**Affiliations:** 1 Department of Rehabilitation Medicine, Bethesda Hospital Yangsan, Gyeongnam Province 626-701, Republic of Korea; 2 Department of Neurosurgery, Bethesda Hospital, Yangsan, Gyeongnam Province 626-701, Republic of Korea; 3 Stem Cell Research Center, K-STEMCELL, 2–305 IT Castle, Gasan-dong, Geumcheon-gu, Seoul 153-768, Republic of Korea

**Keywords:** Autologous adipose tissue-derived mesenchymal stem cell, Cell therapy, Progressive supranuclear palsy, Intravenous infusions, Intrathecal infusions

## Abstract

**Introduction:**

Progressive supranuclear palsy is a relentlessly progressive neurodegenerative disorder and is clinically characterized by parkinsonism. Adipose tissue-derived mesenchymal stem cells have recently demonstrated the possibility of treating neurological disorders. Therefore, autologous adipose tissue-derived mesenchymal stem cells transplantation can be an alternative method for treating progressive supranuclear palsy.

**Case presentation:**

This study was approved by the Korea Food and Drug Administration through the Emergency Use Investigational New Drug Application. A 71-year-old Asian man from South Korea with progressive supranuclear palsy was treated with five intravenous infusions (each time 2×10^8^ cells) and four intrathecal infusions (each time 5×10^7^ cells) with autologous adipose tissue-derived mesenchymal stem cells expanded under good manufacturing practice conditions. Clinical examinations were performed immediately before treatment and throughout the six months of follow-up. The tests included: 1) Progressive Supranuclear Palsy Rating Scale; 2) Berg Balance Scale; 3) Korean Mini Mental State Examination; 4) Modified Barthel Index; 5) grip strength; 6) Box and Block Test; and 7) Nine-Hole Peg Test.

The Progressive Supranuclear Palsy Rating Scale results gradually decreased, and the clinical rating scale scores of the Berg Balance Scale, Korean Mini Mental State Examination, and Modified Barthel Index gradually increased. Grip strength was maintained. Performance in the Box and Block Test and Nine-Hole Peg Test improved after adipose tissue-derived mesenchymal stem cells treatment compared to baseline throughout the six months of follow-up. Except for the intermittent mild fever and transient elevated blood pressure, the treatment of our patient with progressive supranuclear palsy with autologous adipose tissue-derived mesenchymal stem cells showed no significant adverse events, and delayed the progression of neurological deficits by achieving functional improvement in the follow-up period.

**Conclusions:**

These results are encouraging and hopeful for further studies in patients with progressive supranuclear palsy using autologous adipose tissue-derived mesenchymal stem cells as a safe and effective therapy. This case report is the first known study of adipose tissue-derived mesenchymal stem cells safely delaying the progression of progressive supranuclear palsy with functional improvement during the follow-up period.

## Introduction

Progressive supranuclear palsy (PSP) is a late-onset neurodegenerative disease and one of a number of diseases collectively referred to as atypical parkinsonian disorders (APDs) including multiple system atrophy (MSA), dementia with Lewy bodies, and corticobasal degeneration [1,2].

The prevalence of PSP has been estimated at 1.39 to 6.4 per 100,000 [3,4], although this is likely to be an underestimate because of diagnostic confusion with idiopathic Parkinson’s disease based on the clinical similarity of phenotypes such as slow movements and gait difficulty. The clinical features are atypical, and diagnosis is often delayed or not made during the lifespan of the patient. PSP also represents at least 5 percent of patients who manifest parkinsonian symptoms [5]. However, this percentage is probably an underestimate due to the difficulties in diagnosing this syndrome [1,4].

Success in treating patients with PSP remains exceedingly low [6]. Dopaminergic drugs are regularly used for the parkinsonian features, but benefit is poor or transient [1,7,8]. No drug treatment consistently treats PSP patients in the long term, and neuroprotective or regenerative strategies may be invaluable in the management of patients with PSP [6]. Recently, accumulating evidence indicates that mesenchymal stem cells (MSCs) can be an alternative therapy for PSP. The treatment of MSCs had neuroprotective and neuroregenerative effects with idiopathic or APDs in animal [9] and human [10] studies.

Therefore, recent evidence suggests that MSC treatment can be a novel therapy for treating PSP. This is the first case report that describes the efficacy of systemic and intrathecal infusion of autologous adipose tissue-derived MSCs (AdMSCs) for a patient with PSP.

## Case presentation

This study was approved by the Korea Food and Drug Administration with Emergency Use Investigational New Drug Application number CTMD (Clinical Trials Management Division) -2440 and the Institutional Review Board (No. 2012-03-RNL-ASTROSTEM). Prior to stem cell therapy, a consent form was signed by the guardians of the patient and they agreed to provide the medical records for the publication of this report.

An Asian man, 71 years of age during the time of the study, suffered from several disorders of the central nervous system (CNS). When he was 56 years old, he had severe dizziness and vomiting symptoms and was diagnosed with cerebral infarction. At age 58, he had postural instability, leaning to the right side, and was diagnosed with infarction in small vessels of the brain by a magnetic resonance imaging (MRI) scan. In addition, he had urinary incontinence and difficulty with emotional control. When he was 65 years old, he fell on his right side. In that same year, his gait and dysarthria worsened along with his reflex coughing during fluid intake. He was diagnosed with Parkinson’s syndrome and began taking Sinemet® (carbidopa 50mg + levodopa 200mg). After no response, he stopped taking Sinemet®. He received rehabilitation treatment occasionally, yet the symptoms continued. At 71 years of age, he was diagnosed with PSP by a brain positron emission tomography - computed tomography (PET-CT) scan. He took Madopar® (benserazide 50mg + levodopa 200mg) for medication. After no response, he stopped taking the drug. He took aspirin (100mg/daily), Plavix® (clopidogel 37.5mg/daily), and Norvasc® (amlodipine besylate 5mg/daily) starting immediately after the lack of response to medication became apparent and continued during and after the autologous AdMSCs treatments were administered.

Our patient was subjected to hematology and serological tests for liver and renal function prior to the collection of subcutaneous fat from the abdomen via liposuction. His hematology and serological test results were normal. Our patient was not infected with syphilis, human immunodeficiency virus, hepatitis B, or hepatitis C. AdMSCS were isolated, expanded, and analyzed, as previously described in detail [11], according to good manufacturing practice (GMP) conditions. Multiple AdMSC aliquots were prepared at passage 2 and stored in liquid nitrogen vapor. Cryopreserved cells were thawed and recultured in growth medium according to the infusion schedule (see below). Cells were harvested at passage 3, and the quality control tests, including cell viability and for fungal, bacterial, endotoxin, and mycoplasma contamination, were carried out before infusion. No evidence of bacterial, fungal, or mycoplasma contamination was observed.

For 12 weeks, he was treated with five intravenous infusions, each containing 2×10^8^ autologous AdMSCs (1×10^8^ cells/100ml normal saline), via the cephalic vein for about 2 hours (a total of 10^9^ cells via intravenous infusion) [11] and four intrathecal injections, each containing 5×10^7^AdMSCs in 2ml of normal saline, via a standard lumbar puncture (a total of 2×10^8^ cells via intrathecal injection). The patient received rehabilitation therapy after the fourth treatment for 12 weeks.

Safety was monitored extensively on day 0, week 2, week 4, month 2, month 3 and month 6 for tolerance, adverse events, laboratory tests, physical examinations, and vital signs. The clinical improvement was evaluated on the beginning of the first treatment (day 0), and on month 1, month 3, and month 6 of follow-up.

Safety and tolerability were assessed on the basis of hematological and biochemical tests highlighting values outside the normal range, medical examination findings, and adverse event reporting. After AdMSCs infusion, intermittent mild fever (37.8 to 38.5°C) was observed during the second, fourth, and fifth treatments. His blood pressure was elevated to 200/123mmHg with intermittent moderate headache after the second treatment. When his body temperature was over 38°C, Dicknol (diclofenac-β-dimethyl-aminoethanol, 90mg) was administered intramuscularly. Betasin (labetalol 20mg) was injected intravenously for elevated blood pressure. The adverse effects disappeared immediately after Dicknol or Betasin treatment. Except for the intermittent mild fever and the one-time elevation of his blood pressure, there were no significant adverse events during and following autologous AdMSCs treatments.

**Figure 1:**
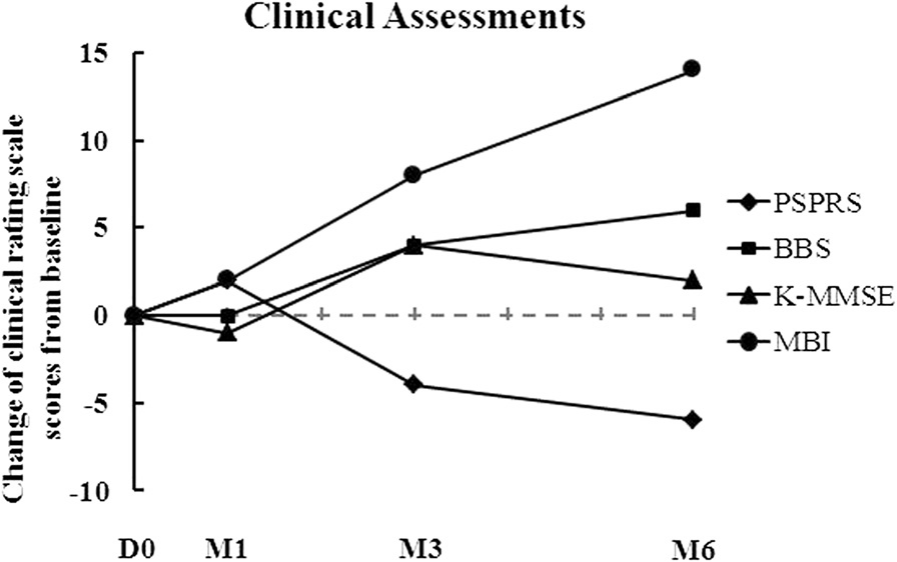
**The change of clinical rating scale scores from baseline throughout the six months of follow-up.** The Progressive Supranuclear Palsy Rating Scale score gradually decreased and the clinical rating scale scores of the Berg Balance Scale, Korean Mini Mental State Examination, and Korean Modified Barthel Index gradually increased. D, day; M, month.

**Figure 2:**
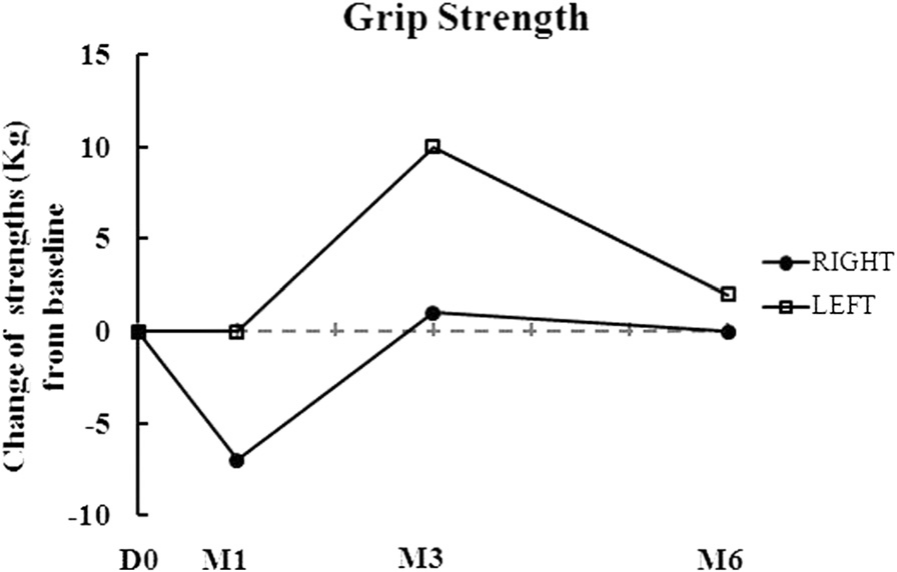
**The change of grip strengths from baseline throughout the six months of follow-up.** The grip strength was maintained after autologous adipose tissue-derived mesenchymal stem cells treatment compared to baseline. D, day; M, month.

**Figure 3:**
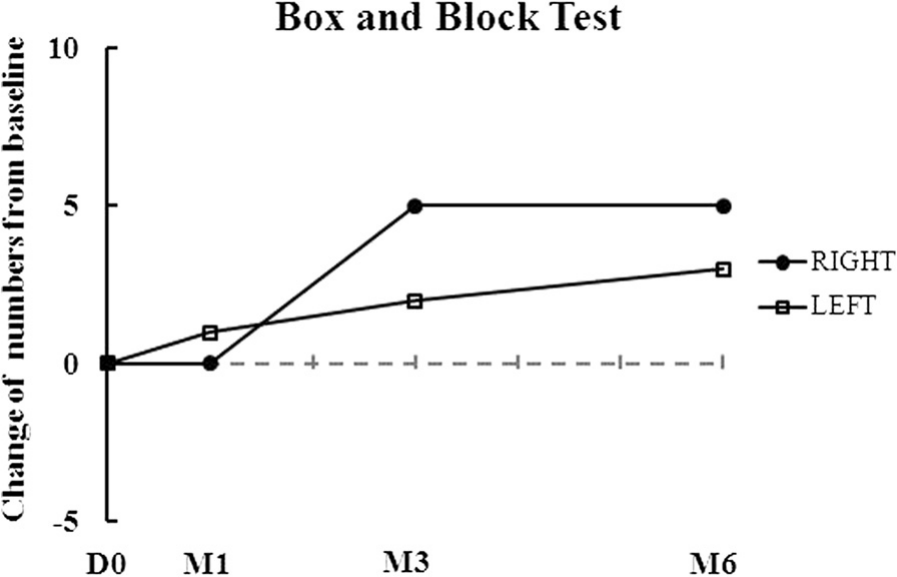
**The change of box and block test (numbers) from baseline throughout the six months of follow-up.** There was improved function of the right hand in the Box and Block Test. D, day; M, month.

**Figure 4:**
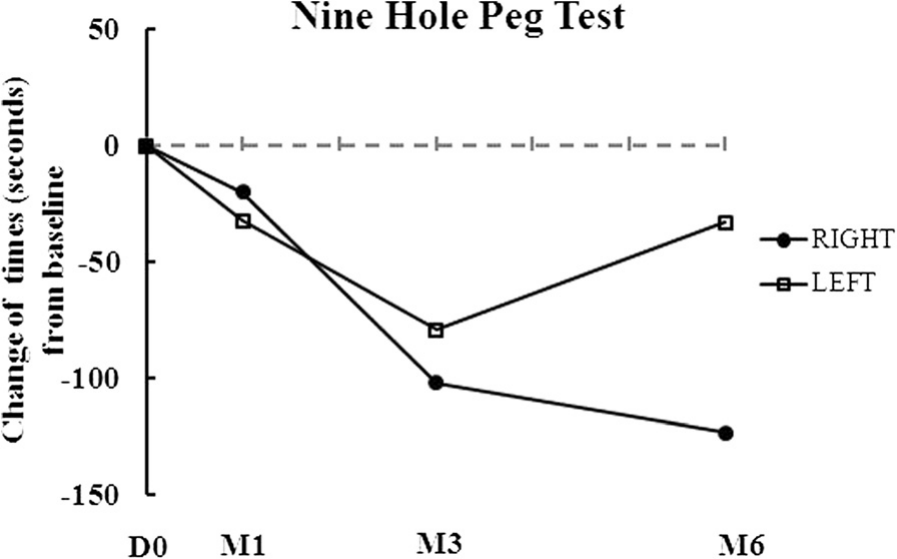
**The change of nine-hole peg test (seconds) from baseline throughout the six months of follow-up.** It was performed faster and the run time was shortened. D, day; M, month.

The results of his clinical assessment are summarized in Table 1 and Figure 1. As shown in Table 1 and Figure 1, the Progressive Supranuclear Palsy Rating Scale (PSPRS) score gradually decreased from 69 to 63 points throughout the six months of follow-up after AdMSC treatment. The clinical rating scale scores gradually increased from 3 to 9 points in the Berg Balance Scale (BBS), from 15 to 17 points in the Korean Mini Mental State Examination (K-MMSE), and from 7 to 21 points in the Modified Barthel Index (MBI) throughout the six months of follow-up after AdMSC treatment compared to baseline (Table 1 and Figure 1). The grip strength, Box and Block Test (BBT), and Nine-Hole Peg Test (NHPT) were also performed to measure the strength or functional performance of the upper limbs. The grip strength was maintained from 8 to 8kg on his right hand and increased from 8 to 10kg on his left hand (Table 1 and Figure 2) after stem cell treatment. His results improved from 9 to 14 numbers of blocks transported on his right hand and from 13 to 16 numbers of blocks transported on his left hand during BBT (Table 1, Figure 3). Throughout the six months of follow-up, in NHPT, he performed faster, and the run time was shortened from 202 to 79 seconds on his right hand and from 127 to 94 seconds on his left hand (Table 1, Figure 4) after AdMSC treatment compared to baseline.

**Table 1:** Clinical assessments between pre- and post-treatments of autologous adipose tissue-derived mesenchymal stem cells (AdMSCs) in a patient with progressive supranuclear palsy (PSP) throughout the six months of follow-up

	**Day 0**	**Month 1**	**Month 3**	**Month 6**
**PSPRS** (/Total 100 points)	69	71	65	63
**BBS** (/Total 56 points)	3	3	7	9
**K-MMSE** (/Total 30 points)	15	14	19	17
**MBI** (/Total 100 points)	7	9	15	21
**Grip strength** (Kg, R/L)	8/8	1/8	9/18	8/10
**BBT** (Numbers, R/L)	9/13	9/14	14/15	14/16
**NHPT** (Seconds, R/L)	202/127	182/95	100/48	79/94

PSPRS, Progressive Supranuclear Palsy Rating; BBS, Berg Balance Scale; K-MMSE, Korean Mini Mental State Examination; MBI, Modified Barthel Index; BBT, Box and Block Test; NHPT, Nine-Hole Peg Test.

## Discussion

PSP is a rare disease with rapidly progressive disabling neurological function and is fatal within 10 years of onset [12,13]. The patients' mean age at onset of PSP is typically between 55 and 65 years with a median survival time of 5.6 to 7.3 years [13]. The natural course of PSP is a relentlessly progressive deterioration. One-year survival has a rate of 70.2 percent but two-year survival has a rate of only 28.5 percent for patients with scores from 60 to 69. One-year survival rate was 47.4 percent but two-year survival rate was 16.4 percent for patients with scores over 70 [12]. There is no significant benefit of current therapies for patients with PSP [12,13].

Recently, accumulating evidence indicates that MSCs can differentiate into neural cells *in vitro*[11] and protect the brain in animal models from CNS injuries such as Alzheimer’s disease [14] or Parkinson’s disease [9,10]. The adipose tissue is a very attractive source for MSCs. It contains a large amount of MSCs and can be easily obtained.

In our animal study of Alzheimer’s disease, intravenously administered human AdMSCs localized in the CNS, differentiated into nerve cells, and protected the system from the death of nerve cells by increasing neuroprotective agents [14]. These results present the possibility of treating patients with PSP with MSCs. In this case report, we used mixed administration routes of intravenous and intrathecal. While the intrathecal route may enhance the therapeutic effects by facilitating AdMSCs movement to the CNS, the dosage of ASCs is limited due to the narrow intrathecal cavity compared to intravenous administration. Therefore, we also used the intravenous route to increase the dosage of AdMSCs, leading to a higher efficacy.

The patient in the present study was diagnosed with infarction of cerebral microvessels at 57 years old and has suffered from CNS disorders for 15 years. In addition, he had had a worsening of symptoms such as gait problems, postural instability, urinary incontinence, emotional problems, and cognitive impairment during the six years since the diagnosis of Parkinson’s syndrome, before it being confirmed as PSP. Our patient was treated with autologous AdMSCs when the disease had progressed to severe status. The safety of the autologous stem cell infusion via both intravenous and intrathecal routes was monitored. Hematological and biochemical tests showed no significant adverse reactions after stem cell infusion. In line with our results, the safety of administration of AdMSCs [11] was reported by demonstrating that none of the patients developed any serious adverse events related to MSC treatment. In regard to the efficacy, after treatment with AdMSCs, the PSPRS score was gradually decreased 6 points from baseline, and cognitive impairment and the activities of daily living were improved throughout the six months of follow-up. Although, our results were for a short period of follow-up of one patient, the multiple intravenous and intrathecal infusions of autologous AdMSCs were safe and effective in delaying the progression of the disease.

The pathophysiological knowledge of brain dysfunction, particularly for Alzheimer’s disease, has increased over the years. Much of this knowledge has been revealed using current imaging techniques along with other genetic and pathological research. This knowledge provides an understanding of the mechanism that leads to brain dysfunction and disease [15]. In the current study, however, we focused the evaluation on the safety rather than efficacy of stem cells due to our conditions as patients with PSP are rare, difficult to diagnosis and revealing of the neuropsychological changes. Nevertheless, to the best of our knowledge, this clinical case is the first case report using adipose tissue-derived mesenchymal stem cells to treat a patient with PSP.

We believe that increasing preclinical and clinical data on MSCs’ effect in conjunction with a more large-scale clinical study on neurodegenerative disease will lead to a more basal understanding of brain damage and neurodegenerative disease.

Taken together, our results with the studies of other clinical researchers present an encouraging treatment of autologous MSCs to those suffering from atypical Parkinson syndrome who have a short prognosis.

## Conclusions

The current results suggest that repeated infusion of autologous AdMSCs can be a safe and effective therapy for PSP. We anticipate that repeated administration of an adequate number of AdMSCs will prevent further neurological damage following the onset of PSP.

In conclusion, this is the first case report showing that autologous AdMSCs treatment of a patient with PSP was safe and delayed the progression of neurological deficits by achieving functional improvement in the follow-up period. At the least, these results are encouraging and hopeful for further studies on more patients with PSP.

## Consent

Written informed consent was obtained from the guardians of the patient for publication of this case report and any accompanying images. A copy of the written consent is available for review by the Editor-in-Chief of this journal.

## Abbreviations

AdMSC: adipose tissue-derived mesenchymal stem cell; APD: atypical parkinsonian disorders; BBS: Berg Balance Scale; BBT: Box and Block Test; CNS: central nervous system; CTMD: Clinical Trials Management Division; GMP: good manufacturing practice; K-MMSE: Korean Mini Mental State Examination; MBI: Modified Barthel Index; MRI: magnetic resonance imaging; MSA: multiple system atrophy; NHPT: Nine-Hole Peg Test; PET/CT: positron emission tomography - computed tomography; PSP: progressive supranuclear palsy; PSPRS: Progressive Supranuclear Palsy Rating Scale.

## Competing interests

Soo Won Choi and Kwon Byong Park have no competing financial interests in this work. Sang Kyu Woo and Sung Keun Kang are employees of K-STEMCELL and declare no competing financial interests. Jeong Chan Ra is an employee and founder of K-STEMCELL.

## Authors’ contributions

SWC was in charge of the patient’s treatment and care and wrote the final manuscript. KBP performed the intrathecal infusions and assisted in manuscript preparation. SKW cultured and ensured the quality control of the AdMSCs. SKK participated in the study coordination and reviewed the manuscript. JCR was involved in the preparation of AdMSCs, directed the coordination of study and managed the study along with the clinicians. All authors read and approved the final manuscript.
